# The Medico-Legal Society

**Published:** 1880-04

**Authors:** 


### THE MEDICO-LEGAL SOCIETY.

*The Problems of Insanity.*—Dr. George M. Beard read a paper on the above subject, in which he considered first, the causes of insanity, and of its increasing prevalence in modern times; second, difficulties in the diagnosis; third, the probabilities of cure; fourth, defects of the present system of treatment in Europe and America; and fifth, the future of insanity and influences that may check the rate of increase. The following is a brief abstract: The speaker said that it was one of the paradoxes of astronomy that the constitution of the sun was best studied when that orb was in an eclipse. It was one of the paradoxes of psychology that the mind was best studied when it was eclipsed by disease. Through insanity we learned of insanity. Pathology was the aid of psychology. Dr. Beard thought that psychology was destined to be the chief of the sciences, and that its study was greatly aided by the study of insanity. The first problem of insanity was how to define it. Insanity was a disease of the brain, in which mental co-ordination was seriously impaired. There might be disease of the brain without mental inco-ordination, but in all cases of insanity disease of the brain was implied. The word mind was used in a broad and generous sense. There was no mathematical dividing-line between sanity and insanity. Sanity shades into insanity like twilight into night, and the different forms and subdivisions shade into each other like the colors of the rainbow. There were two general divisions of insanity—intellectual and emotional. Delusions were not necessary to insanity. In very many cases there were no delusions, simply emotional disturbances. The ideas implied by the terms moral insanity and impulsive insanity were correct and sound. In some cases patients afflicted with what was called moral insanity, developed into forms of insanity in which there were delusions. Insanity with delusions and insanity without delusions ran together constantly, and were intermingled in different cases. The term moral insanity might be an unfortunate one, but the idea was scientific and demonstrable.

A second problem was, why was insanity increasing in frequency? Insanity was a barometer of modern civilization. Though existing in all recorded ages, and among all peoples, and known under various and inconsistent names and superstitions, yet it was rare and had always been rare with the savage, the barbarian, and the partly enlightened. There was no race, no climate, no institution, no environment that could make insanity common save when united with and reinforced by brain work and indoor life. Besides those general differences there were features of the modern and pre-eminently of the nineteenth century civilization that were peculiar to it—unprecedented in history—the printing press, the telegraph, steam power, the sciences and the mental activity of woman. Bringing arithmetic to aid us in our comparative estimate, it seemed quite within the facts to aver that the modern brain must carry and endure tenfold more than the ancient, and without a correlated increase of carrying and bearing force. Insanity was increasing more among the poorer than the higher classes. Civilization ground hardest on the poor, depriving them of the healthful influences of barbarism without the compensating advantages that the higher classes enjoyed. Dr. Beard stated that he had reached that conclusion from years of study of this special subject. A number of years before, while studying the subject of stimulants and narcotics, together with the drinking customs of different nations, he had occasion to study very thoroughly the customs of different nations, as far as could be obtained from accessible sources, works of travel and conversations with travellers, and, making due allowance for sources of error, there was an entire agreement with regard to the variety of allied nervous diseases among savages and barbarians. Quite recently he had studied the subject himself among thousands of negroes upon the island of Beaufort, between Savannah and Charleston. These negroes had been for years isolated, had not advanced a great deal beyond their original ancestors in Africa, and furnished an excellent opportunity for him to study the subject himself. He had investigated the psychology of those people carefully, by the aid of persons who, for years, had lived among them and employed them, and diseases such as neurasthenia, hay fever, symptoms of spinal irritation, and insomnia, to any extent, were not known. They drank to excess, but there was no inebriety.

Poverty made us insane, and insanity made us poor. In England the increase of insanity among the poor during the last forty years had been 300 per cent., while the population increased in the same time only 45 per cent. But stastistics relating to insanity were crammed with elements of terror.

The main defects in this present treatment of the insane in Europe and America were these: First neglect of the early stages. The insane should be treated before they were insane. The practical problem of the future was to educate physicians in the study of insanity so that they should know premonitory symptoms and treat the patients in many instances without taking them to an asylum. The time would come when physicians would treat insanity just as much as they treated typhoid fever; would be able to diagnosticate it in the early stages; would be able to cure it; and, what was still better, would be able to prevent it by treating the conditions which lead to it. In a number of cases under his own observation he had thought of referring the patients to an asylum, but satisfactory results had been obtained by treatment at home. Each case in that respect must be judged by itself. A number of his medical friends, general practitioners, he knew were treating certain forms of insanity very successfully in that way.

The solution of that problem was already going on. Patients in the early and premonitory symptoms were constantly and successfully treated both by neurologists and general practitioners. Even those who were positively insane were treated successfully in that way at home. Second, the error was in depending on simple isolation in asylums without positive medication. The same mistake had been made in inebriate asylums, but was being corrected in them. The third evil was in the use of narcotics and sleep-forcing agents. The fourth error was the crowding together of curable and incurable cases. Three-fourths of the cases in asylums were cured in the first nine months. It was one of the facts of human progress that reforms usually came from outside of institutions that needed to be reformed. Long ago, John Bright, in the House of Commons, declared that all the reforms of English law in favor of justice and mercy had been opposed by the judges.

The diminished proportion of cures for the insane in asylums in recent years was explained partly by this, that hereditary influences were every year growing stronger. Hereditary disease of all kinds was harder to cure and more likely to relapse than any accidental or primary nervous disease. The future of insanity was a part of the future of sociology. Hundreds of millions of people were to occupy this continent, and among them were to be hundreds of thousands of lunatics. The late president of the British Psychological Association predicted that if insanity was to increase in England and Wales as it had increased during the last forty years, there would be 1,250,000 lunatics upon that island in the year 1912.

But the relative increase of insanity might probably be checked somewhat in all civilized countries. First, by inventions which diminished the fractions of modern life. Types of those inventions were the telephone, the type writer, palace cars, and elevated railways in great cities. The inventions of thirty or forty years ago, the telegraph and the like, all tended to increase the friction of life. Second, by the development of the intellect at the expense of the emotions. Insanity was caused not so much by intellectual as emotional activity. Third, improved systems of education, primary and university. The whole system of mental training for the young must be, and would be, revolutionized. Fourth, the successful treatment of the nervous diseases and conditions that lead to insanity as nervous exhaustion, cerebral congestions, hysteria, hypochondria, and the like. In that respect great progress was being, and had been, made. There was also going on improvement in mental hygiene. The better classes of the American people were to-day growing stronger and larger, and now presented some of the best specimens of physique in the world. The criticisms on the defects of the treatment of the insane applied to all countries more or less, but England had the great advantage of supervision of all asylums, public and private, by a central authority, composed partly of physicians, partly of lawyers, and partly of business men. For forty years that commission had been in operation in England, and had given satisfaction. A bill to increase the number and enlarge the powers of the New York State Board of Charities, so that it might fulfill the duties assigned to the English Lunacy Commission was before the Legislature. It ought to be introduced not only in this State, but in other States. It would not solve all the problems of lunacy, but it would be one step in the direction of the solution of some of them. Lunacy reform would not be accomplished by leaps or jumps, but by slow increments.

During the discussion that followed the reading of the paper, there was considerable dissent expressed from some of the views contained in it, and it was voted to call a special meeting for the thorough discussion of the entire subject.

